# Relationship between perception of malocclusion and the psychological impact of dental aesthetics in university students

**DOI:** 10.4317/jced.52157

**Published:** 2015-02-01

**Authors:** Carlos Bellot-Arcís, José-María Montiel-Company, Teresa Pinho, José-Manuel Almerich-Silla

**Affiliations:** 1 Adjunct Professor, Stomatology Department, Faculty of Medicine and Dentistry, University of Valencia, Spain; 2 Post-Doctoral Assistant Professor, Stomatology Department, Faculty of Medicine and Dentistry, University of Valencia, Spain; 3 Assistant Professor of Orthodontics, Instituto Superior de Ciências da Saúde-Norte; Centro de Investigação Ciências da Saúde (CICS), Portugal; 4 Ternured Lecturer, Stomatology Department, Faculty of Medicine and Dentistry, University of Valencia, Spain

## Abstract

Introduction and Objectives: The objectives were to assess the relationship between perceived smile aesthetics and perceived psychological impact as measured by the Psychosocial Impact of Dental Aesthetics Questionnaire (PIDAQ), and their own perception of it using the Aesthetic Component of the Index of Orthodontic Treatment Need (IOTN-AC) and a Visual Analog Scale (VAS); relate the IOTN-AC and VAS to the PIDAQ; and study the predictive capacity of the scales for psychological impact. Material and Methods: A cross-sectional study was conducted in 447 college students in Spain and Portugal (average age 20.4 years, 33.1% men and 66.9% women). The online self-completed surveys used the recently-validated Spanish and Portuguese versions of the PIDAQ to assess the self–reported psychological impact of the students’ dental aesthetics and IOTN-AC and an ad hoc 100 mm VAS for their perception of their dental aesthetics. Results: PIDAQ was linearly correlated with IOTN AC and VAS. Pearson’s coefficient was 0.55 for PIDAQ and IOTN-AC (CI 95% 0.48-0.61) and -0.72 for PIDAQ and VAS (CI 95% -0.66 - -0.76). VAS and IOTN-AC were predictive variables in a linear regression model of the total PIDAQ score. The VAS diagnosed individuals whose dental aesthetics had a self-perceived psychological impact (area under the curve 0.827, CI 95% 0.787-0.868) more precisely than the IOTN-AC (area under the curve 0.742, CI 95% 0. 696-0.788). Conclusions: In adults patients, there is a significant linear relationship between perceived smile aesthetics and self-perceived psychological impact.

** Key words:**Visual Analog Scale, Index of Orthodontic Treatment Need, malocclusion, psychological, aesthetics.

## Introduction

The purpose of most occlusal or orthodontic treatment need indices is to assess the anatomical and esthetic aspects of the malocclusion, ignoring the patient’s own perception of it and its effect on his or her quality of life (1,2). The first index to consider the patient’s own esthetic perception was the Aesthetic Component (AC) of the Index of Orthodontic Treatment Need (IOTN) (3). However, its reliability has been questioned by many authors (4-9).

Researchers have used the Visual Analog Scale (VAS) as it is considered a simpler way to ascertain the patient’s perception of the esthetics of his or her smile and, by some authors, a quicker method with high reproducibility (10-13).

In recent years increasing interest has been shown in questionnaires which provide more information on the oral health-related quality of life of the patients and their esthetic perception of themselves (2,14-16). The Psychosocial Impact of Dental Aesthetics Questionnaire (PIDAQ) is a powerful tool that provides very valuable information on aspects of oral health-related quality of life (OHRQoL) (17). This questionnaire is becoming known around the world and versions in Portuguese, Chinese and Spanish have been validated recently (18-20).

Some authors consider that the negative perception of the aesthetic of the smile, could provide a self-perceived psychological impact in adolescents patients (21). But this relationship has not been studied in adults.

The objectives of the present study were:

- To assess the self-perceived psychological impact of malocclusion in a sample of university students using the PIDAQ and their own perception of malocclusion using the IOTN-AC and a VAS

- To relate these two scales (IOTN-AC and VAS) to the Psychological Impact of Dental Aesthetics Questionnaire (PIDAQ)

## Material and Methods

700 students were randomly selected from the database of University of Valencia (Spain) and the University of Oporto (Portugal)). Sampling took place from November 2012 to January 2013. The students were sent an e-mail explaining the objectives of the study and giving the link to the web page with the questionnaire. An informed consent was obtained from the study participants. The study was approved by the University of Valencia Faculty of Medicine and Dentistry ethics committee. The students were asked to complete the survey alone, without any help. For a prevalence of 50%, an accuracy of ±0.05 and a CI 95%; would have an estimate of the sample size of 385. The expected response rate in previous on-line surveys was 60%.

Out of a total of 700 questionnaires, 447 (63.9%) were accepted. The main reason for non-acceptance was not having answered all the questions. A cross sectional study was conducted in a sample of 447 young college students. By subject area, 31.1% were studying Health Sciences (Medicine and Dentistry), 38.1% Social Sciences (Business Management and Administration, Law and Political Science) and 30.6% Engineering. The mean age of the sample was 20.4 years (CI 95% 20.0-20.8), the range was between 18.0 and 30.2 years (median 20.2 years). By gender, 33.1% were men and 66.9% women. The surveys were self-completed online by students of the University of Valencia (Spain) (50.3%) and the University of Oporto (Portugal) (49.7%).

The respondents had to state their age and gender, their degree course subject area (major) and whether they had received orthodontic treatment. Those who were wearing orthodontic devices at the time of the study were excluded, but not those who had been treated in the past. The online questionnaire contained the PIDAQ, IOTN-AC and VAS. It took approximately 8 minutes to complete.

The PIDAQ (Psychosocial Impact of Dental Aesthetics Questionnaire), recently validated for use in Spanish and Portuguese, was employed to assess the self-reported psychological impact of dental aesthetics (17-20). The Aesthetic Component (AC) of the Index of Orthodontic Treatment Need (IOTN) was used to assess the perception of dental aesthetics (3). An ad hoc 100 mm Visual Analog Scale (VAS) was also included. The IOTN AC scale of 10 black and white photographs was shown with the explanation: “These ten photographs show different levels of dental attractiveness. Number 1 is the most attractive and number 10 the least attractive. Where on this scale would you place your teeth?” The VAS was explained as follows: “This is a scale to assess how you perceive your dental aesthetics. Taking the left end as “very bad or least attractive” and the right end as “very good or most attractive”, use the cursor to mark where on the scale you would place how you think your teeth look.” The right end was labeled with a smiley face and the left end with a sad face. The VAS score was obtained by measuring the distance in millimeters between the mark made by the student and the far left of the scale.

The means and confidence intervals of the questionnaire and the two scales were then calculated. Pearson’s correlation coefficients were employed to study the relations between them. Linear regression models were used to assess the predictive capacity of IOTN-AC and VAS for the self-perceived psychological impact of the malocclusion. Their diagnostic precision was measured by ROC curves. Following the validation study, the PIDAQ score (≥36) of the individuals with orthodontic treatment need was taken as the gold standard (20).

To assess the reproducibility of the method, a pilot study was first carried out in 30 individuals who self-completed the questionnaire twice, one month apart. The results showed high reproducibility in all three, PIDAQ, IOTN-AC and VAS (intra-class correlation coefficients of 0.94, 0.90, and 0.96 respectively). The data were analyzed by the SPSS program (19.0).

## Results

The mean PIDAQ score was 47.1 (CI 95% 45.5-48.7). The mean IOTN-AC score was 1.88 (CI 95% 1.77-1.99). The mean VAS score was 72.9 (CI 95% 71.1-74.8).

The PIDAQ results showed no differences by gender or nationality but did display differences by subject area. The engineering and social science students’ scores were significantly higher (*p*<0.05) than those of the health sciences students ( Table 1). On the IOTN-AC scale the men scored more highly than the women (*p*<0.05), there were no differences by nationality but the health sciences students’ scores were significantly lower ( Table 1). The VAS scores exhibited differences by gender, nationality and subject area ( Table 1).

**Table 1:**
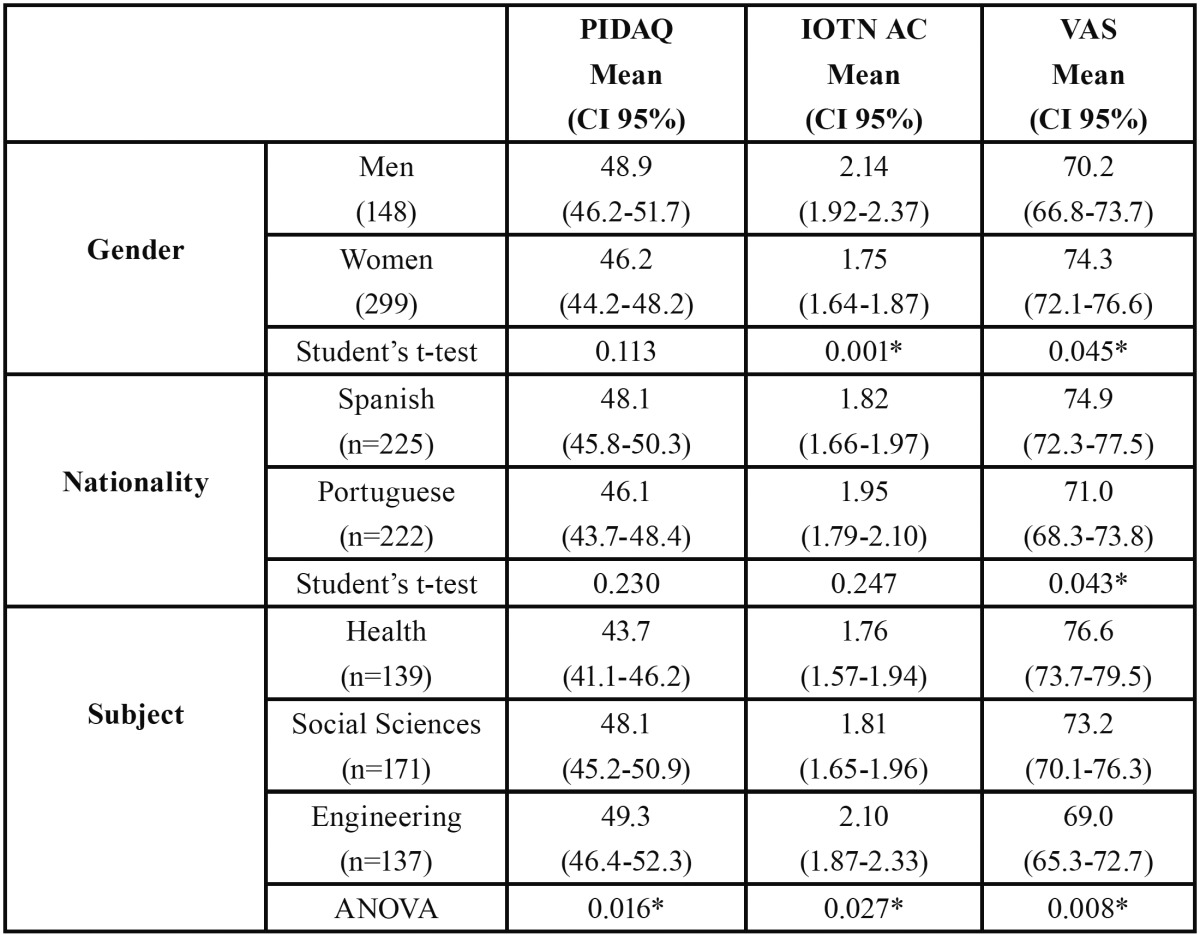
PIDAQ, IOTN-AC and VAS by gender, nationality and subject area. Student’s t-test and ANOVA: *p<0.05

The results showed linear correlation between PIDAQ, IOTN-AC and VAS. Pearson’s correlation coefficient was 0.55 between PIDAQ and IOTN-AC (CI 95% 0.48-0.61), -0.72 between PIDAQ and VAS (CI 95% -0.66 - -0.76) and -0.52 between IOTN-AC and VAS (CI 95% -0.45 - -0.59).

Both VAS and IOTN-AC are predictive variables in a linear regression model of the total PIDAQ score ( Table 2). No influence of gender or age was found.

**Table 2:**
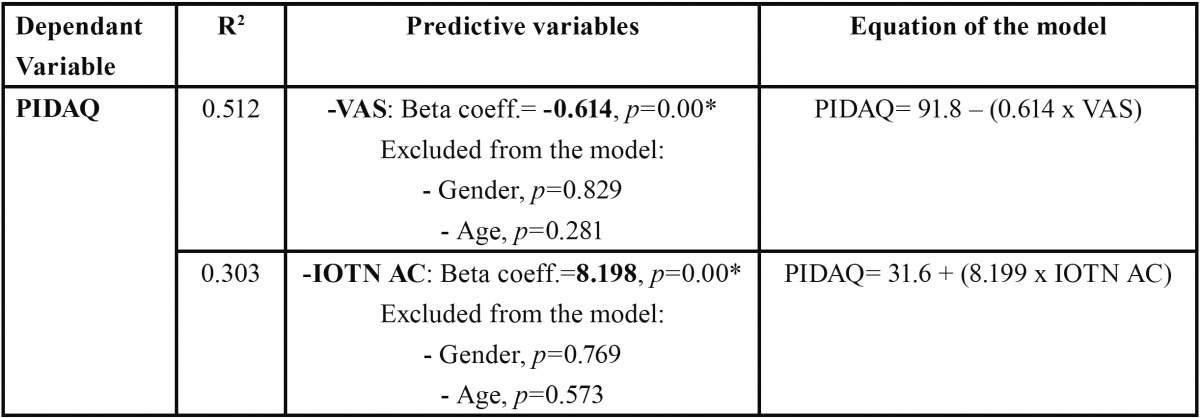
Predictive models of PIDAQ from VAS and IOTN AC.

**Figure 1:**
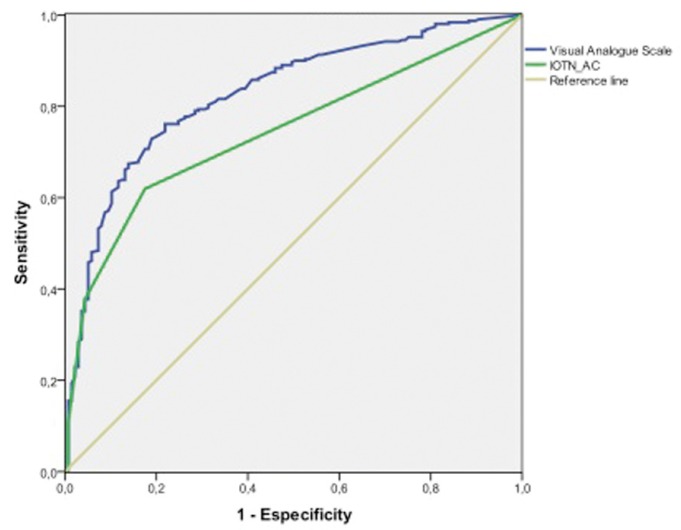
ROC curve: diagnostic precision of VAS and IOTN AC for psychological impact of malocclusion.

The VAS was more precise than the IOTN-AC in diagnosing the individuals whose dental esthetics had a self-perceived psychological impact (respectively area under the curve 0.827, CI 95% 0.787-0.868, and area under the curve 0.742, CI 95% 0. 696-0.788) (Fig. 1).

## Discussion

One of the main disadvantages of studies based on online questionnaires is answer bias, although the reproducibility results of the pilot study were satisfactory. Self-completed questionnaires preserve anonymity and the fact of being online and quickly answered encouraged completion. Even so, 33% of those who accessed the web page left it without completing the items and were excluded from the study, which made it difficult to obtain a larger sample. Another difficulty in designing the survey in the online format was how to include a VAS.

Concerning the age of the sample, the decision was taken to conduct the study in young adults (college students) because many authors have concluded that it is better to analyze the self-perceived psychosocial impact of dental aesthetics in adults, as they already possess a certain emotional stability and have a more realistic view of dentofacial aesthetics (22-25).

The aesthetic perception of the smile is not the same than the self-perceived psychological impact it may cause, but our study shows that the two concepts are strongly related in the young adult population.

The results showed high linear correlation between the self-reported psicological impact using PIDAQ, and the self-perception aesthetics using IOTN-AC or VAS. Pearson’s correlation coefficient was 0.55 for PIDAQ and IOTN-AC. The coefficient for PIDAQ and VAS was negative (-0.72) because the VAS was measured in the opposite direction to the AC, which gives the lowest score to the most attractive photo.

In linear regression models, both VAS and IOTN-AC demonstrated their capacity to predict the PIDAQ score. However, the VAS was able to predict 51% of the variability in the model and the IOTN-AC only 33%. On comparing them through ROC curves, VAS again showed greater diagnostic precision than IOTN-AC.

We consider that for studying the relationship between aesthetic self-perception of malocclusion and its self-perceived psychological impact, is not necessary the use of objective indices such as DAI or IOTN DHC. The psychological impact of malocclusion just depends on patient’s self-perception, and not on the measurements obtained by objective indices. The purpose of the orthodontic treatment need indices is to assess the occlusal and esthetic aspects of the malocclusion, ignoring the patient’s own perception of it and its effect on his or her quality of life (1,2). Moreover many authors have attempted to develop methods or indices to analyze the patients’ perceptions of their dentofacial esthetics. This was the aim of the IOTN-AC, which has indeed been widely used (26). However, its reliability has been questioned by many authors (4,6-9). The authors of the present study have observed in previous research that patients sometimes hesitate between two very different photographs, showing the difficulty they have in identifying with any of the ten photographs (27). Other studies have found that results differ considerably according to whether the patient’s need is measured objectively with the IOTN DHC or with the IOTN AC (9,28).

Also, it should not be forgotten that the IOTN-AC shows ten frontal photographs but patients do not normally see their teeth in this way. Nevertheless they are asked to use these photographs for comparison. Moreover, there are as many smiles as there are people and it makes no sense to limit the patient’s choice to only ten. In addition, many aspects that do not appear in the IOTN-AC are overlooked (such as anterior crossbite), and increased overjet, despite being of considerable concern to patients, is undervalued, as it is not evident in a frontal photograph (29).

For these reasons, it was decided to use the VAS (Visual Analog Scale) as a complementary method, as it is considered a clear, simple way to assess the patient’s esthetic perception and has been used by several authors (10-12,30). Some researchers highlight its simplicity and ease of use and the fact that the absence of numbers or images reduces the possibility of bias (31).

In summary, in adults patients, there is a significant linear relationship between self-perceived smile aesthetics and self-perceived psychological impact.
